# Gene expression profiling in cardiac tissue of mice chronically infected with *Toxoplasma gondii*: implications for immune response pathways

**DOI:** 10.1186/s40001-025-03088-z

**Published:** 2025-08-26

**Authors:** Marzieh Asadi, Mohammad Hossein Banabazi

**Affiliations:** 1 Department of Parasitology, Toxoplasmosis Research Center, Communicable Diseases Institute, Mazandaran University of Medical Sciences, Sari, Iran; 2 Department of Medical Parasitology and Mycology, School of Medicine, Kerman University of Medical Sciences, Kerman, Iran; 3 Department of Animal Biosciences (HBIO), Centre for Veterinary Medicine and Animal Science (VHC), Swedish University of Agricultural Sciences (SLU), 75007 Uppsala, Sweden; 4 Department of Biotechnology, Animal Science Research Institute of IRAN (ASRI), Agricultural Research, Education & Extension Organization (AREEO), 3146618361 Karaj, Iran

**Keywords:** *Toxoplasma gondii*, Chronic infection, Heart, Pathway analysis, Rt-PCR

## Abstract

*Toxoplasma gondii* is a common and opportunistic protozoan in vertebrates. Most cases of infection are asymptomatic, but in individuals with an immune system deficiency, it can cause severe complications. The parasite has the ability to form cysts in the host’s heart, brain, and other tissues. Heart tissue damage during toxoplasmosis infection is associated with many complications, such as myocarditis, pericarditis, arrhythmia and heart failure. The parasite can survive and create a chronic form of infection by changing the expression of genes effective in the immune system. Therefore, the present study aims to investigate expression dynamics of candidate genes in the heart tissue of mice infected with the chronic *T. gondii* strain of Tehran and the pathways involved. After causing toxoplasmosis infection in mice and confirming the infection by MAT, RNA was extracted from the heart tissue of mice and converted into cDNA. Then, real-time PCR was performed to check the expression changes based on the designed primers of the target genes, and pathway analysis was performed. The results of real-time PCR analysis showed that all candidate genes have a higher expression in infected heart tissue compared to non-infected heart tissue. In addition, gene ontology and pathway analysis revealed that the cytosolic DNA-sensing pathway and autoimmune thyroid illness had the most gene participation.

**Graphical Abstract:**

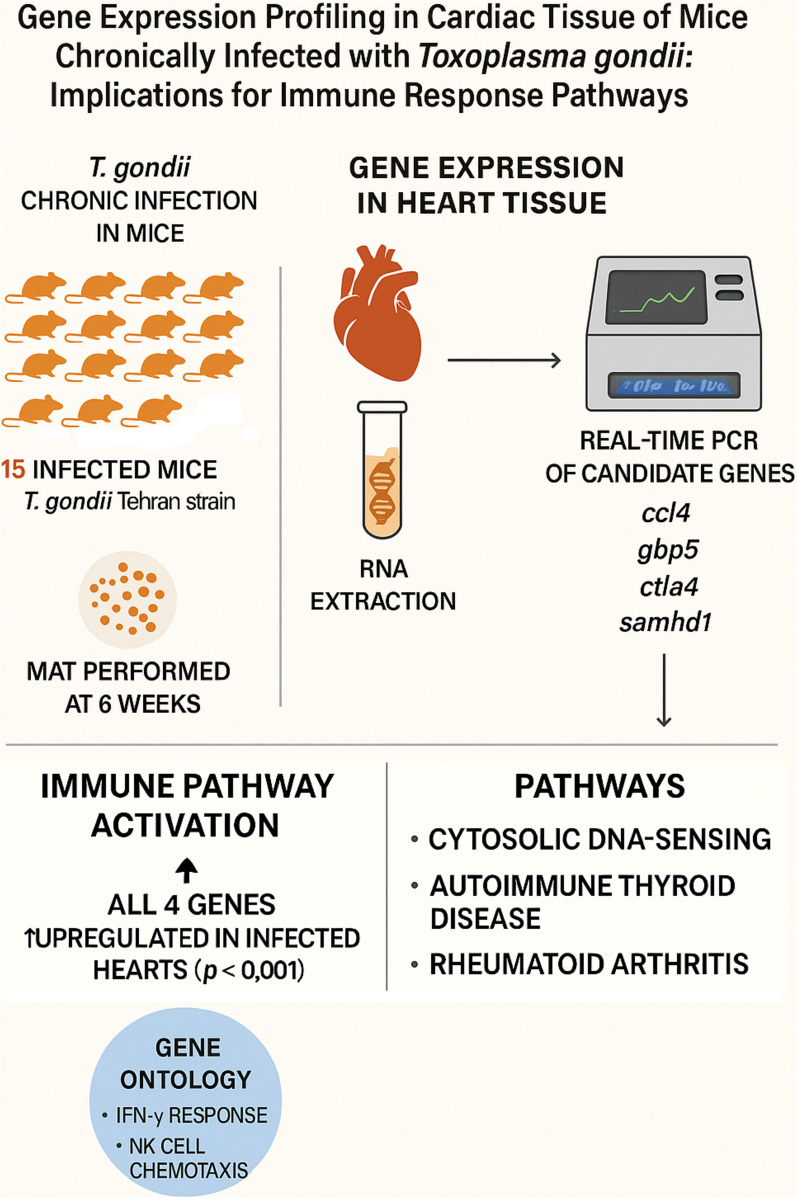

**Supplementary Information:**

The online version contains supplementary material available at 10.1186/s40001-025-03088-z.

## Introduction

*Toxoplasma gondii* is an obligate intracellular protozoan from the Apicomplexa phylom that causes toxoplasmosis [1]. The infection caused by this parasite has infected 25–30% of the world’s population [2, 3]. In the life cycle of *T. gondii*, three infectious stages have been identified, including sporozoite, tachyzoite and bradyzoite [4]. Transmission occurs predominantly via ingestion of the tissue cysts, ingestion of oocysts [5, 6].

*T. gondii* infection is associated with the parasite overpowering the host’s immune responses [7, 8]. In the acute form of infection, tachyzoites multiply rapidly in the nucleated cells of the host and after cell lysis, they are released and spread to other cells [9].

*T. gondii* overcomes immune responses and uses them to transform tachyzoites into bradyzoites and cause chronic infection in susceptible tissues as a source of permanent infection [10]. The chronic form of infection is associated with the formation of parasite cysts. *T. gondii* cysts are formed in many tissues, including: brain, liver, lung, heart, kidney, muscle, lymph nodes, pancreas, bladder and skin [11].

Studies have shown that chronic *T. gondii* infection can be associated with heart disease. *T. gondii* has been identified as the second protozoan agent associated with myocarditis. For the first time, parasite cysts were identified and described by Kean and Grocott in the myocardium. In previous studies, infection with *T. gondii* has been identified as a factor associated with cardiac injuries, including myocarditis (inflammation of the heart muscle), constrictive pericarditis, pericardial effusion, atrial and ventricular arrhythmias, and acute heart failure. Although cardiac involvement due to toxoplasmosis is generally rare and often asymptomatic or masked by neurological symptoms, there is evidence linking this parasite to cardiac dysfunction and cardiovascular mortality. Reactivation of *T. gondii* infection can exacerbate cardiac damage and diastolic heart failure, as observed in animal models. In addition, reports exist of acute myocarditis and transient thickening of the heart muscle in animals infected with this parasite. Despite this, diagnosing *toxoplasmic* myocarditis remains challenging and often requires endomyocardial biopsy. Serological and molecular studies are widely used for infection diagnosis.

Therefore, existing evidence suggests that *T. gondii* infection can lead to serious cardiac complications in some patients [12].

Cardiac damage caused by *T. gondii* infection depends on the extent of the host’s inflammatory reactions in such a way that during infection, the greater the intensity of the inflammatory reactions, the greater the damage to the heart tissue [13, 14]. Myocarditis is the most common and pericarditis is the least clinical manifestation of toxoplasmosis in the heart. The symptoms of patients with toxoplasmic myocarditis include arrhythmia congestive heart failure and constrictive pericarditis [15, 16].

After the detection of *T. gondii* by the host’s innate immune cells, cytokines such as IL-12, TNF-*α* and IL-6 are produced, and the level of IFN-*γ* increases with the activation of the acquired immune response [17].

*T. gondii* requires modulation of the immune system and regulation of related signaling pathways of the host’s immune system to maintain its survival [18]. The parasite can escape from being eliminated by immune cells in different tissues, including the heart, with clever strategies and changes in the expression of important genes related to immune responses, and the infection can become chronic, thus maintaining its survival in the host [17, 18].

Therefore, assessing the gene expression changes in the heart tissue of mice infected with the chronic form of *T. gondii* infection can be valuable for clarifying the reactions of the parasite and the host and the pathways involved. According to the mentioned cases, the present study aims to investigate of expression dynamics of candidate genes *ccl4, gbp5, ctla4*, and *samhd1* in the heart of mice infected by a chronic *T. gondii* Tehran strain, to investigate the gene ontology and with to analyze the pathways involved in the genes.

## Materials and methods

### Ethics statement

The code of ethics of this study was IR.KMU.REC.1399.548 which was registered in Kerman University of Medical Sciences. Keeping animals and working with them was done according to the protocol of working with laboratory animals, and animals were kept in suitable light, temperature, seasonal and nutritional conditions. In this study, all efforts were made to minimize animal pain and distress during chronic infection. Animals were monitored daily for weight, behavior, and stress, with analgesics and anesthetics used as needed. Procedures were performed by trained personnel to reduce harm, and the study duration was kept as short as possible. Severe pain cases were managed promptly with humane interventions. The study followed the 3Rs principles and complied with national and international animal care guidelines.

### Development of chronic toxoplasmosis in animal models

Tehran strain was used to create a chronic form of toxoplasmosis infection. A number of 20 parasite cysts were injected into the peritoneum of 15 Balb C male mice aged 5 weeks and weighing 35 g to create a chronic form of infection.

To confirm infection in Balb C mice, the modified agglutination test (MAT) was performed at 6 week post-injection. This timepoint was selected to capture the transition from the acute to the chronic phase of toxoplasmosis, during which tissue cysts form and stabilize in the heart. In the acute phase, an initial immune response characterized by elevated IgM and IgG antibodies peaks within a few weeks. By 6 weeks, the infection progresses to the chronic stage with persistent cysts and ongoing inflammation. Therefore, sampling at this stage allows for the assessment of molecular and gene expression changes reflective of chronic infection and its long-term effects on cardiac tissue.

The kit used in this test was BIOMERIEUX, and by observing direct agglutination, positive samples were detected in terms of contamination. The control group (15 Balb C mice) was injected with a normal saline solution and matched with the group infected with *T. gondii* in terms of age and sex. This matching was performed to reduce confounding variations and increase the accuracy of comparisons.

### Extraction of RNA and cDNA synthesis

After euthanizing the mice in both the case and control groups, the heart samples of the mice were separated for experiments.

First, RNA was extracted from heart tissues using a commercial kit RNeasy mini kit (Qiagen, Chatsworth, CA, USA) according to the manufacturer’s protocol, and evaluated using nanodrop (Nano Drop ND-1000, Thermo Scientific, Wilmington, DE, USA). Suitable samples were kept at − 70 degrees until the cDNA synthesis. Then cDNA was synthesized using a Takara Prime Script^™^ RT reagent kit (Takara Bio, Inc., Japan).

### Real-time PCR

To determine the expression of genes *ccl4, gbp5, ctla4*, and *samhd1*, real-time PCR was performed. After the synthesis of cDNA, the desired primers were designed and the amplification of the desired parts of the genes was carried out using the rotor-gene cycler system (rotor-gene 3000 cycler, Corbett, Sydney, Australia) and SYBR Green master mix (SYBR Premix Ex Taq^™^ II, Takara Bio, Inc., Shiga, Japan). The temperature and time program of the real-time PCR reaction includes initial denaturation for 1 min at 95 °C, 40 three-step cycles (denaturation: 10 s at 95 °C, primer annealing: 15 s at 58 °C, extension: 20 s at 72 °C) and the final elongation for 1 min at a temperature of 65 °C.

GAPDH gene was also used for internal control. Statistical analysis of gene expression was performed using the ΔΔCt method. Each sample was analyzed in triplicate, and results were expressed as mean ± standard deviation. Statistical significance between groups was assessed using unpaired student’s *t* tests, with a significance threshold set at *p* < 0.001. GraphPad Prism v9.0 was used for all analyses and visualizations. The sequence of primers used is given in Table 1.

**Table 1:** Genes and primers used in real-time PCR

Gene	Accession no.	Primer name	Primer sequence (5′–3′)	Product length (bp)
*ctla*4	NM-005214.5	CTLA4-F CTLA4-R	CTGCAAGGTGGAGCTCATGTA GGGCACGGTTCTGGATCAAT	90
*ccl*4	NM-002984.4	CCL4-F CCL4-R	CGCAGCCAGCTGTGGTATTC ACAGGATTCACTGGGATCAGC	70
*gbp*5	NM-052942.5	GBP5-F GBP5-R	AGCTATCGACCTACTGCACAATG AAGTCTGGGAAGAAGCTCGC	70
*samhd*1	NM-001363733.2	SAMHD1-F SAMHD1-R GAPDH-F: GAPDH-R:	CTCTGTGTTCAGATTGCTGGACT TGAGCCTTGTTCATGCGTCC 5′-AAGGTCATCCCAGAGCTGAA-3′ 5′-CTGCTTCACCACCTTCTTGA-3′	123

### Gene ontology analysis

The role of genes in molecular, cellular and biological functions as well as the pathways involved was investigated by site https://maayanlab.cloud/Enrichr/.

## Results

The results of the MAT assay showed that a significant percentage of the samples tested positive. In addition, the RNA concentration extracted from the heart tissue of infected and non-infected mice was accurately and reliably measured using a nanodrop device, indicating the high quality of the extracted RNA. In this study, the expression changes of *ccl4, gbp5, ctla4,* and *samhd1* genes were investigated after performing real-time PCR.

**Fig. 1:**
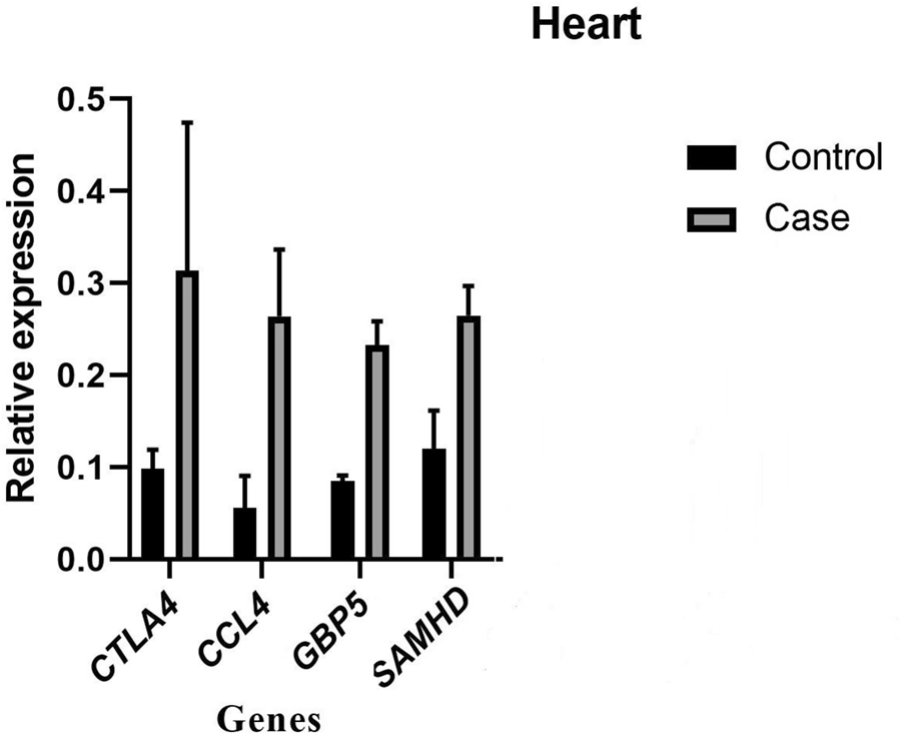
* A Significance level of *p* < 0.001 was considered

The results of real-time PCR analysis showed higher expression of all genes in infected heart tissues compared to non-infected ones (Fig. 1).

**Fig. 2:**
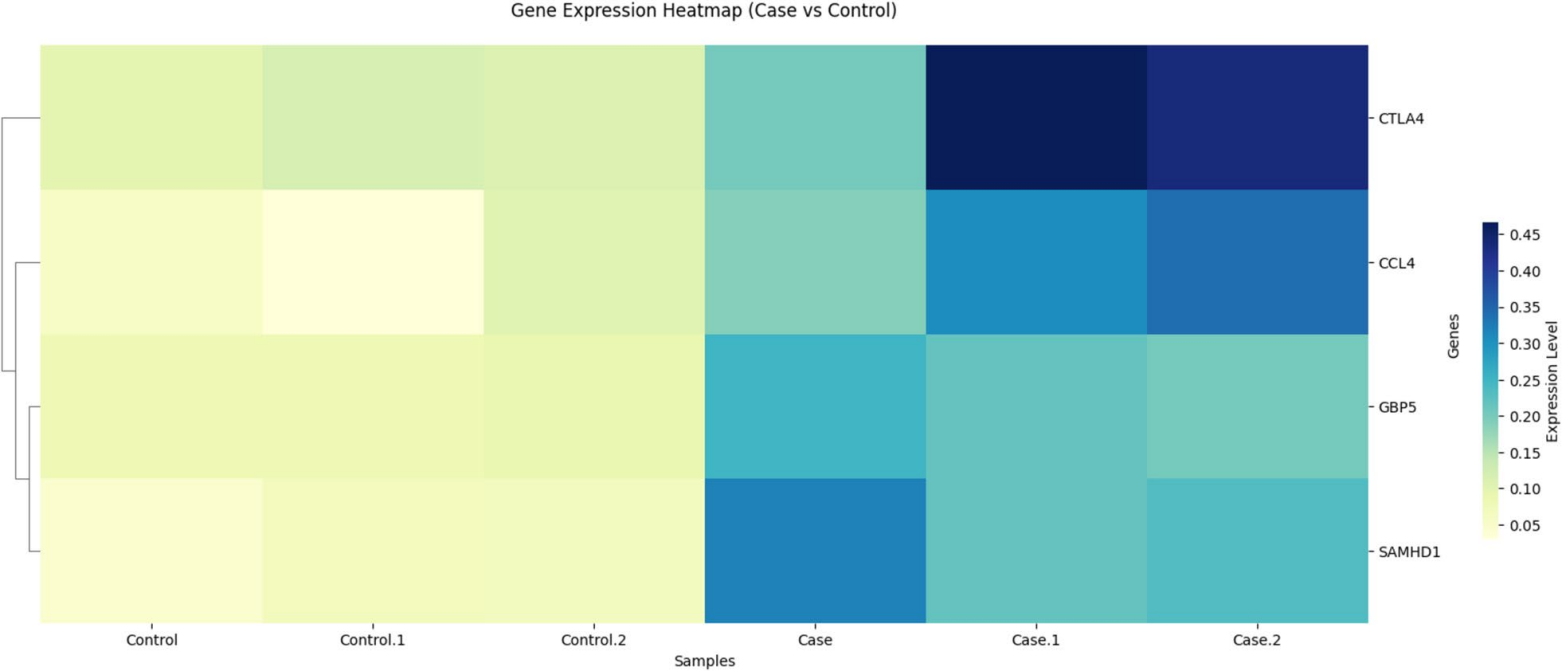
Heat map showing the change in the expression of the desired genes in case and control samples

In addition, these results were shown by heatmap (Fig. 2). In infected samples, *ctla4* gene had the highest expression and *gbp5* gene had the lowest expression.

### Gene ontology

**Fig. 3:**
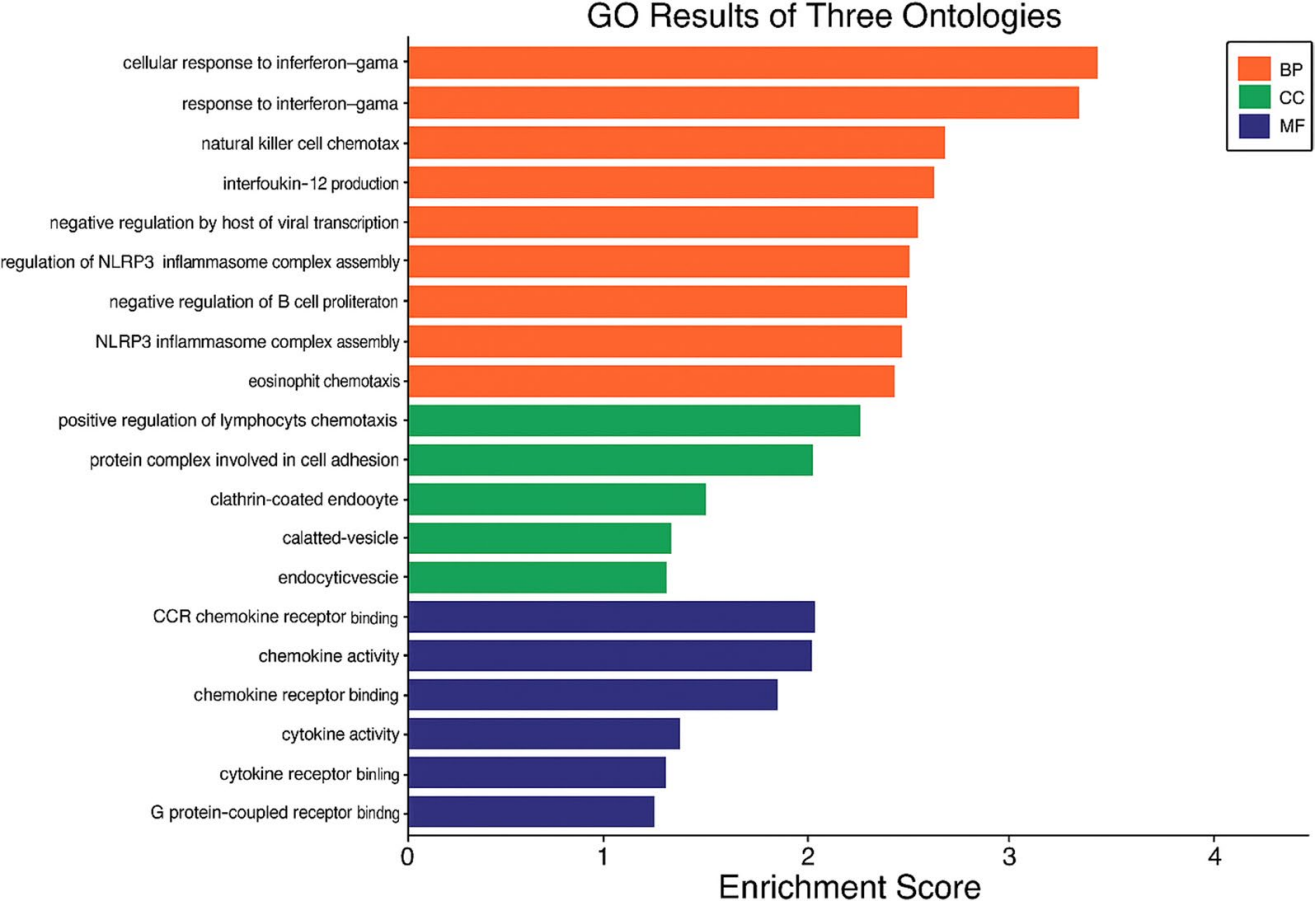
Overview of gene ontology analysis of genes in biological processes (BP), cellular component (CC) and molecular functions (MF)

Gene ontology analysis shows the participation of genes in various molecular, biological and cellular processes, Fig. 3.

As shown, the most participation of genes in biological processes is in cellular response to interferon-gamma, response to interferon-gamma and natural killer cell chemotaxis.

The most participation of genes in cellular components is in protein complex involved in cell adhesion and clathrin-coated endocytic vesicle and also the most participation of genes in molecular functions is shown in CCR chemokine receptor binding, chemokine activity and chemokine receptor binding.

**Fig. 4:**
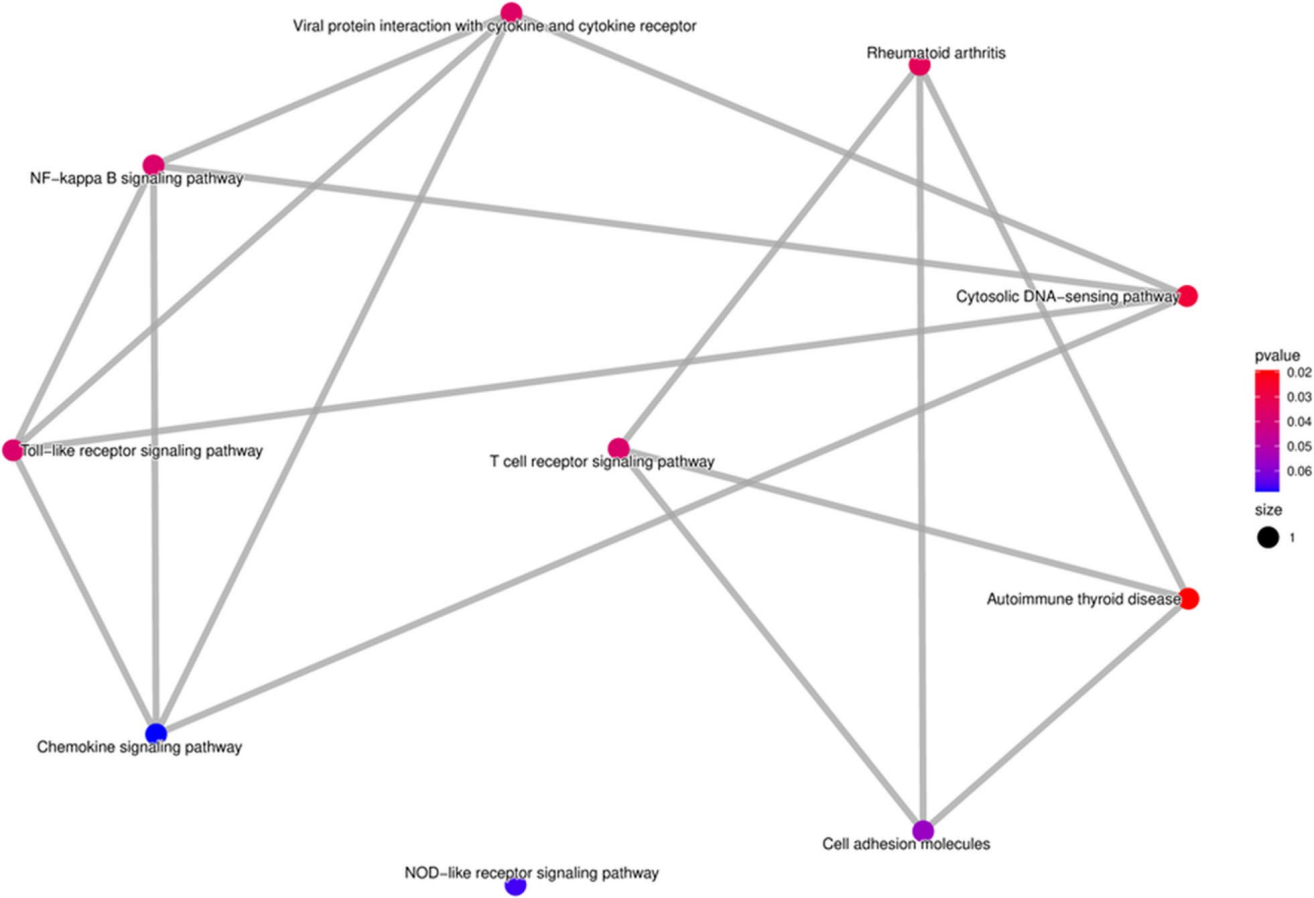
Emapplot: pathways analysis of genes

**Fig. 5:**
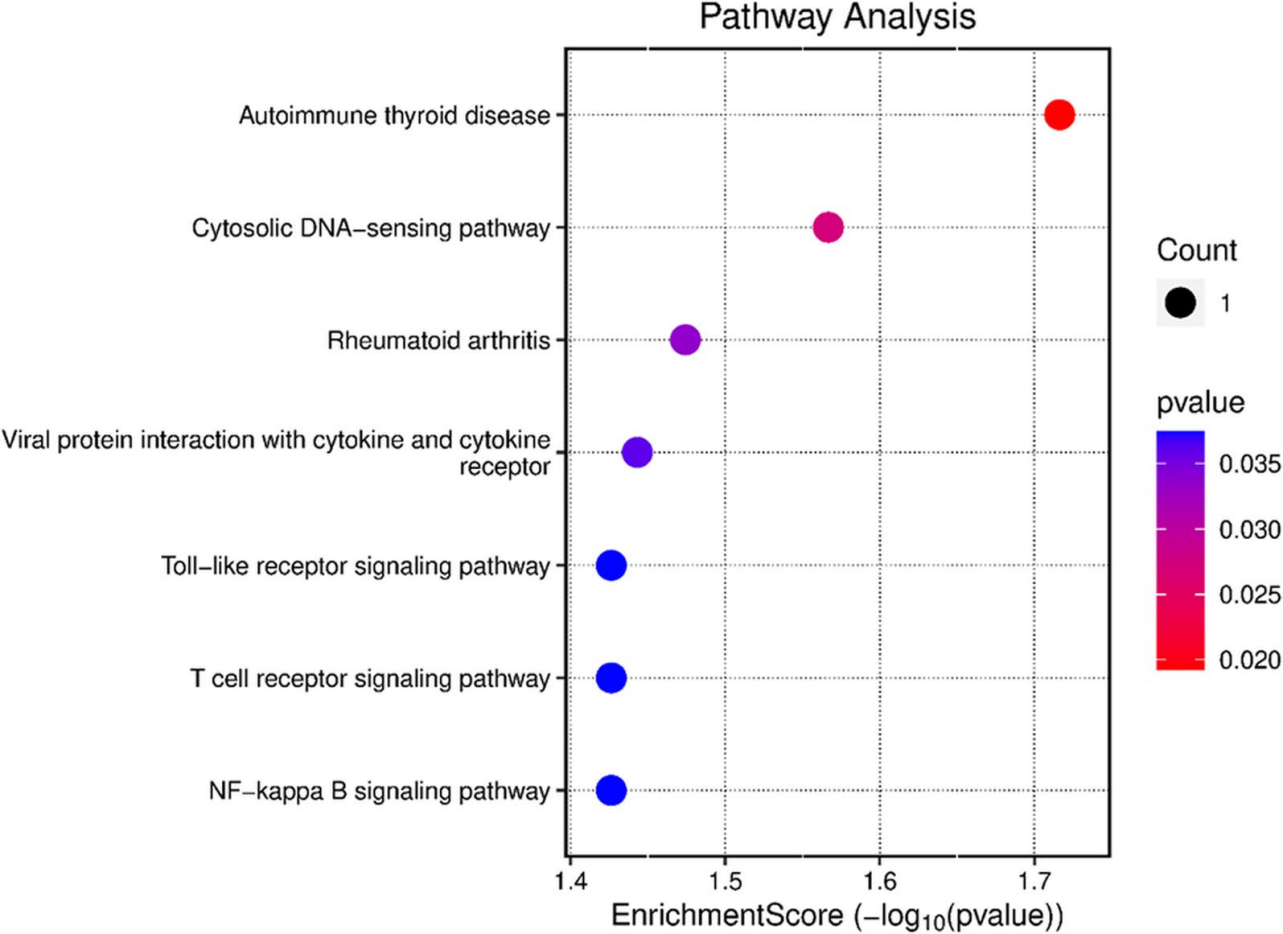
Dotplot: pathways analysis of genes

Pathway analysis has shown that the highest participation of genes is involved in the autoimmune thyroid disease, cytosolic DNA-sensing pathway, and rheumatoid arthritis pathways, Figs. 4 and 5.

## Discussion

The chronic form of *T. gondii* infection is the result of the ability and success of the parasite to escape from the host’s immune system [8]. By modulating the reactions of the immune system, as well as changing the expression of genes effective in the chronicity of the infection, the parasite causes its long-term survival in the host [19].

*T. gondii* is able to cause systemic infection and can remain in muscles such as heart muscle [15]. Toxoplasmosis myocarditis is caused by the involvement of the heart with the parasite, as well as the result of the involvement of organs such as the brain and lungs with the parasite [20]. Sometimes, in very rare cases, death due to toxoplasmic myocarditis has also been reported. Toxoplasmosis can affect gene expression in the mouse heart through several mechanisms. First, direct infection of cardiac cells by the parasite *T. gondii*, which is capable of entering these cells and forming cysts, can lead to changes in the regulation of genes related to cellular function, immune response, and oxidative stress in heart tissue. In addition, *Toxoplasma* infection activates the immune system and increases the production of inflammatory cytokines, such as TNF-*α* and IL-6; these cytokines alter gene expression in cardiac cells through paracrine and autocrine signaling, causing inflammation and tissue damage. Moreover, the parasite induces oxidative stress and metabolic changes in cardiac cells, which can activate or inhibit molecular signaling pathways, thereby affecting the expression of genes involved in cell survival, apoptosis, and tissue repair. Furthermore, toxoplasmosis can indirectly influence gene expression and heart function by producing enzymes that affect the synthesis of neurotransmitters like dopamine. Therefore, through a combination of direct effects on cardiac cells, activation of inflammatory responses, and metabolic and neural alterations, toxoplasmosis can modify gene expression in the mouse heart, ultimately impacting the function and health of cardiac tissue [2, 15, 21].

The results of real-time analysis showed that selected genes effective in the chronicity of toxoplasmosis have a higher expression in the heart tissue of infected mice compared to non-infected ones.

In this study, an increase in C–C motif chemokine ligand 4 (*CCL4*) gene expression was observed in the case group compared to the control group. The high expression level of *ccl4* gene in the chronic form of infection plays an important role in the transfer and control of immune cells to the sites of infection [22]. The factors secreted by the parasite, during the chronic form of infection, stimulate the production of interleukin 12 from dendritic cells and macrophages, as well as the interaction between chemokines, such as CCL4, CCL5 and CCL3 [23].

In addition, when the immune system is exposed to tachyzoites, the release of NETs (neutrophil extracellular trap) inhibits the tachyzoites and this type of infection leads to chronicity [24]. In addition, in the study of Bonfa et al. in 2014, it was shown that the induction of CCL3, CCL4 and CCL5 genes and pro-inflammatory cytokines (IL-12 and TNF) increased the recruitment of CCR5 + cells to the site of infection caused by *T. gondii*, which is capable of infection. Control and update [23].

The results of this study showed an increase in Guanylate binding protein 5 (*GBP5*) gene expression in infected heart tissue compared to non-infected ones. The gbp gene plays a key role in interferon-gamma-induced cellular defense mechanisms that target intracellular pathogens and plays a major role in controlling *T. gondii* infection. *gbp1*, *gbp 2* and *gbp 5*are growth inhibitors of *T. gondii* [25].

Induction of *gbp* by interferon gamma helps to create a chronic form of infection by regulating inflammation following *T. gondii* infection [26]. In a study conducted by Saeij et al. in 2017, the main role of *gbp* in host defense against *T. gondii* was shown that tryptophan catabolism, which is an important limiting pathway of IFNγ, interacts with autophagy and *gbp* [27].

The results of the study by He et al., which focused on transcriptomic changes in the liver tissue of mice infected with *T. gondii*, are fully consistent with the findings of this study in heart tissue and reinforce the overall picture of the host’s systemic response to this parasitic infection. Both studies observed increased expression of the genes *gbp5* and *ccl4*, which play key roles in the host immune response. They demonstrate that *T. gondii* infection leads to widespread activation of immune and inflammatory pathways, accompanied by elevated cytokine expression, resulting in immune cell infiltration and tissue damage. Specifically, in liver tissue, critical metabolic pathways such as energy metabolism, fatty acid metabolism, and pathways related to bile synthesis and secretion were significantly downregulated, indicating functional impairment caused by chronic inflammatory responses. In addition, decreased expression of key transcription factors like PPAR, which are important regulators of metabolism and inflammation control, was observed. This concordance of results highlights the significance of immune responses and metabolic alterations induced by toxoplasmosis across different host organs and suggests that *T. gondii* infection can systemically impair function and cause damage in vital tissues, such as the heart and liver. These findings not only confirm the alignment between the two studies but also emphasize the importance of *gbp5* and *ccl4* as molecular markers of immune response and potential therapeutic targets in toxoplasmosis infection [21, 28–30].

The expression of SAM and HD domain containing deoxynucleoside triphosphate triphosphohydrolase 1(*samhd1*) gene plays an active role in chemokine guiding pathways of the immune system [31]. The *samhd1* gene is a regulator of the interferon signaling pathway and the innate immune response, and samhd1 protein interacts with several key proteins in the cellular signaling pathway are able to inhibit inflammation and innate immune response against *T. gondii* infection and lead the infection to become chronic [32].

In this study, the comparative examination of the candidate genes of heart tissue infected with parasites compared to healthy heart tissue, showed the increased expression of *samhd1* gene in infected heart tissue compared to healthy heart tissue, which highlights the role of increased expression of this gene in the chronicity of infection.

Immune response cells such as regulatory *T* cells participate in immune modulation, and surface molecules such as cytotoxic *T* lymphocyte associated *ctla4* develop an immunosuppressive role and can contribute to the spread of infection [33].

The results of this study showed an increase in the expression of* ctla4* gene in infected heart tissue compared to the control group. *ctla4* gene induces indoleamin2,3-di oxygenase1,2 (IDO1, IDO2) enzymes. The induction of these enzymes destroys the amino acid tryptophan. The parasite needs this enzyme for its active reproduction. Therefore, increasing the expression of *ctla4* gene can prevent the proliferation of the parasite and create a chronic form of infection [34]. In a study conducted by Splitt SD et al. in 2018, the expression of *ctla4* was shown in the peritoneum of mice infected with *T. gondii* [33]. The results obtained in this study are consistent with the findings of Pittman et al., who conducted research on the brain tissue of mice infected with *T. gondii*. The expression of the *ctla4* Gene in regulatory *T* cells (Tregs) not only acts as a suppressive factor but also plays a key role in maintaining the homeostasis and stability of these cells’ function. This mechanism demonstrates the complex interaction through which Tregs utilize *ctla4* Gene to control the immune response and regulate *Toxoplasma* infection [35].

## Conclusion

The results of this study clarify the role of candidate genes in contributing to the development of a chronic form of infection during infection with *T. gondii* type II (Tehran strain) in heart tissue. It is hoped that the results of this study will add to the depth of the knowledge of parasite and host reactions and will help in understanding more of the immune pathways involved and as a result design new drugs.

## Supplementary Information

Additional file 1.

## Data Availability

No datasets were generated or analysed during the current study.
